# Continuous positive airway pressure therapy might be an effective strategy on reduction of atrial fibrillation recurrence after ablation in patients with obstructive sleep apnea: insights from the pooled studies

**DOI:** 10.3389/fneur.2023.1269945

**Published:** 2023-11-09

**Authors:** Feng Li, Chang-Jian He, Chun-Hua Ding, Ru-Xing Wang, Hui Li

**Affiliations:** ^1^Department of Cardiology, The Second Affiliated Hospital of Soochow University, Suzhou, Jiangsu, China; ^2^Department of Cardiology, Aerospace Center Hospital, Beijing, China; ^3^Department of Cardiology, The Affiliated Wuxi People’s Hospital of Nanjing Medical University, Wuxi People’s Hospital, Wuxi Medical Center, Nanjing Medical University, Wuxi, China

**Keywords:** continuous positive airway pressure, obstructive sleep apnea, atrial fibrillation, ablation, recurrence

## Abstract

**Background:**

Obstructive sleep apnea (OSA) is an independent and modifiable risk factor in the initiation and maintenance of atrial fibrillation (AF). However, the effective of the continuous positive airway pressure (CPAP) on AF patients with OSA after ablation is elusive.

**Methods:**

Cochrane Library, PubMed, Embase, and Web of Science were systematically searched up to February 1, 2023. Studies comprising the AF recurrence rate between the CPAP therapy group and non-CPAP therapy group for the AF patients with OSA were included. Meanwhile, trial sequential analysis (TSA) was conducted to adjust the lower statistical power and random error in this study. Subgroup analysis identified the potential determinants for the AF recurrence rate with CPAP therapy.

**Results:**

A total of eight studies including 1,231 AF patients with OSA were eligible. Compared with non-CPAP treatment group, CPAP treatment group was statistically associated with a lower AF recurrence rate (risk ratio [RR], 0.58; *p* = 0.000). TSA indicated the firm evidence favoring CPAP group for AF recurrence risk. Three significant intervention-covariate interactions for AF recurrence was identified, including study design, non-paroxysmal AF (PAF) proportion, and CPAP treatment strategy.

**Conclusion:**

Our study suggests that CPAP therapy might be an effective strategy on reducing AF recurrence post-ablation for AF patients with OSA. The CPAP treatment strategy and the non-PAF proportion might be the possible determinants on AF recurrence for AF patients with OSA after ablation.

**Clinical trial registration:**

https://www.crd.york.ac.uk/prospero/display_record.php?ID=CRD42023398588, identifier (CRD42023398588).

## 1. Introduction

Atrial fibrillation (AF) is a commonly and clinically sustained arrhythmia, with an epidemiologically estimated prevalence of more than three million patients in the US, which could lead to stroke, heart failure, impaired quality of life, and increase of hospitalizations (1). Ablation therapy, such as radiofrequency (RF) or cryoballoon (Cryo) ablation, is an effective strategy for rhythm control for drug-refractory and symptomatic AF patients (2). Obstructive sleep apnea (OSA), a growingly common form of sleep-disordered breathing, is characterized by the repeated upper airway collapse during sleep, subsequently causing to sleep fragmentation and oxygen desaturation (3).

Accumulated studies demonstrated that OSA is an independent and modifiable risk factor for cardiovascular diseases, especially in the initiation and maintenance of AF, which was reportedly attributed to multiple abnormal pathophysiologies, including inflammation, oxidative stress, vascular endothelia dysfunction, and sympathetic overactivity (4–7). Moreover, OSA was also an key predictor for the AF prognosis post-ablation, suggesting that OSA could significantly influence the success rate of AF treatment (8). Therefore, the latest perspective for AF management recommended that the optimal management for OSA should be performed to reduce AF symptoms, AF progression, and AF recurrences (9).

CPAP has been considered to be an effective alternation to markly improve and correct the abnormal pathophysiologies underlying the OSA (10, 11). Growing evidence demonstrated that the CPAP therapy could significantly reduce the AF episodes in OSA patients (12, 13). However, the effective of the CPAP on AF patients with OSA after ablation is still controversial. The previous studies and meta-analysis have indicated that CPAP treatment on the AF patients with OSA could decline the risk of AF recurrence after ablation (14, 15). Whereas, a recent randomized controlled trial (RCT) showed the negative results (16).

Therefore, we perfomed this registered meta-analysis to clarify the role of CPAP on the AF patients with OSA after ablation, and identify the potential determinants for the AF recurrence rate between the CPAP therapy group and non-CPAP therapy group for the AF patients with OSA.

## 2. Methods

### 2.1. Study design

This study was performed following the preferred reporting items for reviews and the PRISMA guidelines. The official protocol was registered on the PROSPERO database (CRD42023398588).

### 2.2. Search strategy

Two reviewers (F. Li and C-J. He) independently and comprehensively searched for four databases, including PubMed, Embase, Web of Science, and Cochrane Library, from the establishment of the online databases up to 1 February 2023. The searching keywords mainly included “atrial fibrillation,” “AF,” “obstructive sleep apnea,” “OSA,” “continuous positive airway pressure,” “CPAP,” “ablation,” and “recurrence.” The detailed search strategy was presented in the Supplementary Text 1. Trails comprising the outcomes of AF ablation (mainly AF recurrence rate) between the CPAP therapy group and non-CPAP therapy group for the AF patients with OSA were included. A manual search was also conducted to screen the potential publications not being identified previously. Moreover, the relevant corresponding authors were contacted to acquire the missing data related outcomes in their publications.

### 2.3. Study selection

Two reviewers (F. Li and C-J. He) comprehensively and independently reviewed full texts for screening the eligible studies, respectively. The eligible study would be confirmed if inclusion criteria were met: (1) RCT and observational studies, (2) studies comprising the AF recurrence rate between the CPAP therapy group and non-CPAP therapy group for the AF patients with OSA were included, and (3) studies with the most data of multiple publications for the same study. Studies review articles, editorials, letters, case reports, animal studies, and without original data were excluded. A third reviewer (H. Li) would discuss and resolve the potential disagreements on the eligibility.

### 2.4. Data extraction and quality assessment

Two researchers (C-J. He and F. Li), respectively, acquired the data from all eligible studies. Any controversies were consulted and resolved by a third one (R-X. Wang). First, we documented the eligible study characteristics, including publication year, the first author, study design, the sample size in the CPAP therapy group and non-CPAP therapy group, and follow-up time. Then, the study demographic and clinical characteristics, as well as the intervention-related indexes were also recorded.

A total of two appraisal tools were applied by two independent researchers (F. Li and C-J. He) to evaluate the quality of the eligible studies. For observational studies, the Newcastle-Ottawa Quality Assessment Scale (NOS), including three domains with nine points, was used. The study quality was divided into low quality (the total score < 6) and moderate-to-high quality (the total score ≥ 6). For randomized clinical trial included in this study, the Cochrane risk of bias assessment tool was used, providing a grade of risk of bias for the eligible study in multiple aspects of the study design (such as the selection bias, the performance bias, the detection bias, the attrition bias, and the reporting bias). Any potential controversies were resolved by a third one (C-H. Ding).

### 2.5. Statistical analysis

In this study, continuous variables were displayed as means ± standard deviations or median with interquartile range, and categorical variables were presented as frequencies or percentages. Relative risk (RR) and corresponding 95% confidence interval (CI) were calculated for the AF recurrence rate for all eligible studies. The Stata 16.0 was used for analyses. Statistically significant was defined as *p* < 0.05.

We utilized the I-squared (I^2^) and chi-squared test to quantify and evaluate the statistical heterogeneity of studies. If the I^2^ value was ≥50% and/or *p* < 0.05 for the chi-squared test, we defined that the between-study heterogeneity was substantial, and a random-effect model would be performed. Otherwise, a fixed-effect model was performed. Trial sequential analysis (TSA) is a useful method providing the required information size (RIS). We performed TSA with TSA viewer (version 0.9.5.10 Beta) to adjust the random error and lower statistical power potentially caused by the limited number of trials in the meta-analysis (17). We set the type I and II errors, respectively, to 5 and 20% (80% power). Sensitivity analysis was conducted by sequentially omitting one study at a time to evaluate the effect of a single study on the overall risk. Potential publication bias was also assessed using Egger’s test.

Additionally, subgroup analysis was conducted to screen the sources of heterogeneity, as well as the potential determinants for the AF recurrence rate between the CPAP therapy group and non-CPAP therapy group for the AF patients with OSA. Based on the study characteristics, previously reported factors and some potential factors, a total of eight subgroup factors were identified, including study design (single-center, observational study vs. RCT), CPAP group sample size (> 50 vs. ≤ 50), follow-up time (> 12 months vs. ≤ 12 months), CPAP treatment strategy (first ablation then CPAP vs. first CPAP then ablation), ablation strategy (PVI only vs. PVI plus), ablation energy (RF only vs. RF/Cryo), the non-PAF proportion (> 50% vs. ≤ 50%), and the mean age (≥60 years vs. <60 years).

## 3. Results

### 3.1. Study selection and quality assessment

**Figure 1:**
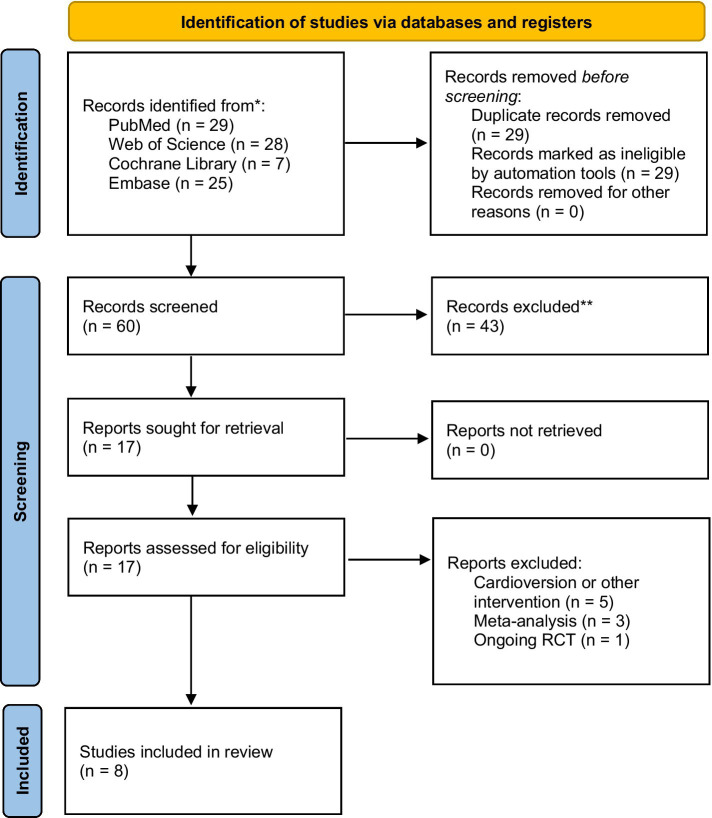
The flowchart for selecting the eligible studies (According to the PRISMA 2020 flow diagram).

Eight studies with 1,231 AF patients with OSA (628 in the CPAP therapy group and 603 in non-CPAP therapy group) were eligible, including seven observational studies (15, 18–23) and one RCT study (16). The selection flowchart was presented in Figure 1. The baseline characteristics and intervention-related indices of the eligible studies were displayed in Table 1.

**Table 1:** The patients characteristics and procedure-related indexes of the eligible studies.

First author	Year	Study design	Sample size		Age (years)		Gender (male, %)		Hypertension (%)		Diabetes mellites (%)	
			CPAP group	Non-CPAP group	CPAP group	Non-CPAP group	CPAP group	Non-CPAP group	CPAP group	Non-CPAP group	CPAP group	Non-CPAP group
Zhou (15)	2022	Single-center, observational study	62	60	59.8 ± 13.4	62.3 ± 9.7	72.6	76.7	96.8	98.3	52.9	61.7
Hunt (16)	2022	RCT	37	46	62.0 ± 8.0	62.0 ± 7.0	76.0	80.0	38.0	39.0	8.0	4.0
Hojo (18)	2019	Single-center, observational study	11	23	66.3 ± 9.6	68.3 ± 8.8	72.7	82.6	72.7	69.6	27.3	30.0
Fein (19)	2013	Single-center, observational study	32	30	56.8 ± 1.2	58.5 ± 1.4	76.7.0	71.9	70.0	65.6	20.0	18.6
Naruse (20)	2013	Single-center, observational study	82	34	NA	NA	NA	NA	NA	NA	NA	NA
Neilan (21)	2013	Single-center, observational study	71	71	NA	NA	NA	NA	NA	NA	NA	NA
Patel (22)	2010	Single-center, observational study	315	325	49.0 ± 8.0	53.0 ± 12.0	71.0	77.0	53.0	69.0	13.0	23.0
Jongnarangsin (23)	2008	Single-center, observational study	18	14	NA	NA	NA	NA	NA	NA	NA	NA

First author	Non-PAF (%)		AADs (% or number of AADs)		LAD (mm)		LVEF (%)		Follow-up (months)
	CPAP group	Non-CPAP group	CPAP group	Non-CPAP group	CPAP group	Non-CPAP group	CPAP group	Non-CPAP group	
Zhou (15)	95.1	96.7	37.1	38.3	45 ± 2.7	44 ± 3.5	52 ± 5.9	50 ± 7.3	12.0
Hunt (16)	0.0	0.0	II: 57%; IV: 3%; I: 27%	II: 57%; IV: 9%; I: 30%	25.2 ± 4 m2	24.5 ± 4.9 m^2^	56.7 ± 3.6	57.1 ± 4.5	12.0
Hojo (18)	27.3	13.0	NA	NA	41.5 ± 6.2	41.7 ± 7.4	63.6 ± 10.3	62.4 ± 14.1	23.3
Fein (19)	56.7	50.0	1.47 ± 0.12	1.34 ± 0.10	54.5 ± 0.91	55.9 ± 1.10	60.2 ± 1.5	59.5 ± 0.94	12.0
Naruse (20)	NA	NA	NA	NA	NA	NA	NA	NA	18.8
Neilan (21)	NA	NA	NA	NA	41.0 ± 6.0	44.0 ± 6.0	56.0 ± 9.0	55.0 ± 10.0	42.0
Patel (22)	52.0	67.0	14.0	30.0	40.0 ± 6.0	50 ± 8.0	50.0 ± 6.0	48.0 ± 5.0	32.0
Jongnarangsin (23)	NA	NA	NA	NA	NA	NA	NA	NA	7.0

First author	OSA diagnosis definition	CPAP treatment	CPAP treatment strategy	Ablation strategy	Ablation energy		Atrial arrhythmia recurrence definition
					CPAP group	Non-CPAP group	
Zhou (15)	The diagnosis of OSA was made by six channels cardiorespiratory polygraphy. OSA was defined as cessation of airflow for more than 10 s with persistent respiratory effort as seen in the ribcage or abdominal motion and complemented by at least 4% fall in O_2_ saturation. An apnea hypopnea index >15/h with at least 80% of all events obstructive was needed for the definition of OSA.	CPAP use at least 5 h per night and for a minimum of the follow-up duration	First ablation then CPAP	PVI only	RF 100%	RF 100%	Atrial arrhythmia recurrence was defined as any documented atrial tachyarrhythmia episode lasting for >30 s 2 weeks after the ablation, was observed at 12 months post-ablation.
Hunt (16)	Moderate to severe OSA was defined by an apnea-hypopnea index (AHI) of 15 events in each hour on the respiratory polygraph test.	CPAP use at least 4 h per night	First CPAP then ablation	PVI only	RF 46%	RF 26%	The implantable loop recorders (ILR) were utilized to detect AF with standard mode via analyzing the incoherence and irregularity of RR intervals and *P* waves during 2 min intervals.
Hojo (18)	OSA was diagnosed as an apnea-hypopnea index (AHI) more than 15	Patients adherent to CPAP treatment	First ablation then CPAP	PVI plus	RF 100%	RF 100%	The arrhythmias were assessed according to the patient’s symptoms, as well as a resting 12-lead electrocardiogram during regular visits to our outpatient clinic. To detect atrial tachyarrhythmia (AT), we performed a 24 hours Holter monitoring at 1 month, 3 months, and 6 months after the first treatment session, and at 1 month, 3 months, 6 months, 12 months, 18 months, and 24 months after the second treatment session. AF and AT recurrent was defined as documented tachycardia lasting >30 s.
Fein (19)	OSA was defined as cessation of airflow for more than 10 s with persistent respiratory effort as seen in the ribcage or abdominal motion and complemented by at least 4% fall in O_2_ saturation. An apnea hypopnea index >15/h with at least 80% of all events obstructive was needed for the definition of OSA.	CPAP use daily for a minimum of 3 months prior to the index PVI, then CPAP use throughout the follow-up duration.	First CPAP then ablation	PVI plus	RF 100%	RF 100%	Arrhythmia recurrence was considered as any documented atrial tachyarrhythmia episode lasting for more than 30 s that occurs after a 2 weeks blanking period following the ablation procedure.
Naruse (20)	The diagnosis of OSA required an AHI of more than 5/h, with more than 50% of the events determined to be obstructive rather than central.	Erratic usage of CPAP as less than 4 h of CPAP use per night throughout the study period	First CPAP then ablation	PVI plus	RF 100%	RF 100%	The presence of AF or any other atrial tachyarrhythmias lasting >30 s after a blanking period of 3 months, off all antiarrhythmic drug therapies
Neilan (21)	Sleep apnea diagnosis with formal sleep studies was meeting the sleep study criteria, which was recommended by the American Academy of Sleep Medicine	Treated OSA was considered as CPAP duration more than 4 hours/night.	NA	PVI plus	RF or Cryo	RF or Cryo	AF recurrence was determined with ECG or prolonged cardiac monitoring more than 3 months after PVI.
Patel (22)	An apnea hypopnea index >15/h with at least 80% of all events obstructive was needed for the definition of OSA.	CPAP use was performed for a minimum of 3 months before the ablation procedure and continued treatment throughout the follow-up period.	First CPAP then ablation	PVI plus	RF 100%	RF 100%	During the first 5 months after ablation, AF recurrence was evaluated using cardiac event monitoring. Patients were asked to transmit their rhythm status three times a day and when they had symptoms consistent with AF. In addition, two-day Holter monitoring was performed at 3 months, 6 months, 9 months, and 12 months and every 6 months thereafter.
Jongnarangsin (23)	OSA was diagnosed based on previously described criteria (PMID: 10450601), without the detail diagnosis criteria.	CPAP use for OSA patients prior to the ablation procedure	First CPAP then ablation	PVI plus	RF 100%	RF 100%	The patients were informed to contact a nurse if symptoms possible suggestive of an arrhythmia occurred. A thirty-day auto-trigger event recorder was provided to patients who remained in normal sinus rhythm at 3 to 6 months post-ablation.

CPAP, continuous positive airway pressure; AF, atrial fibrillation; PAF, paroxysmal atrial fibrillation; AADs, antiarrhythmic drugs; LAD, left atrial diameter; LVEF, left ventricular ejection fraction; RCT, randomized controlled trial; OSA, Obstructive sleep apnea; PVI, pulmonary vein isolation; RF, radiofrequency; PVI plus, PVI plus linear ablation or/and substrate ablation; Cryo, cryoablation; NA, not available.

The ablation energy was RF in two studies (16, 21) while that was RF/Cryo in the remaining six studies (15, 18–20, 22, 23). The ablation strategy of PVI plus was performed in six studies (18–23) while the PVI only was performed in the remaining two studies (15, 16). A total of seven eligible studies reported the CPAP treatment strategy (including first ablation then CPAP (15, 18) and first CPAP then ablation (16, 19, 20, 22, 23)) while one study (21) did not. Except for the Jongnarangsin et al. study (23) (only 7 months follow-up), the remaining seven studies (15, 16, 18–22) have a more than 12 months follow-up. Moreover, the non-PAF proportion was available only in five eligible studies (15, 16, 18, 19, 22), and the remaining three studies did not reported the proportion of AF type (20, 21, 23). All non-RCT studies showed a moderate-to-high quality (Supplementary Table S1). The RCT had a relatively high quality (Supplementary Table S2).

### 3.2. The AF recurrence between CPAP group and non-CPAP group

**Figure 2:**
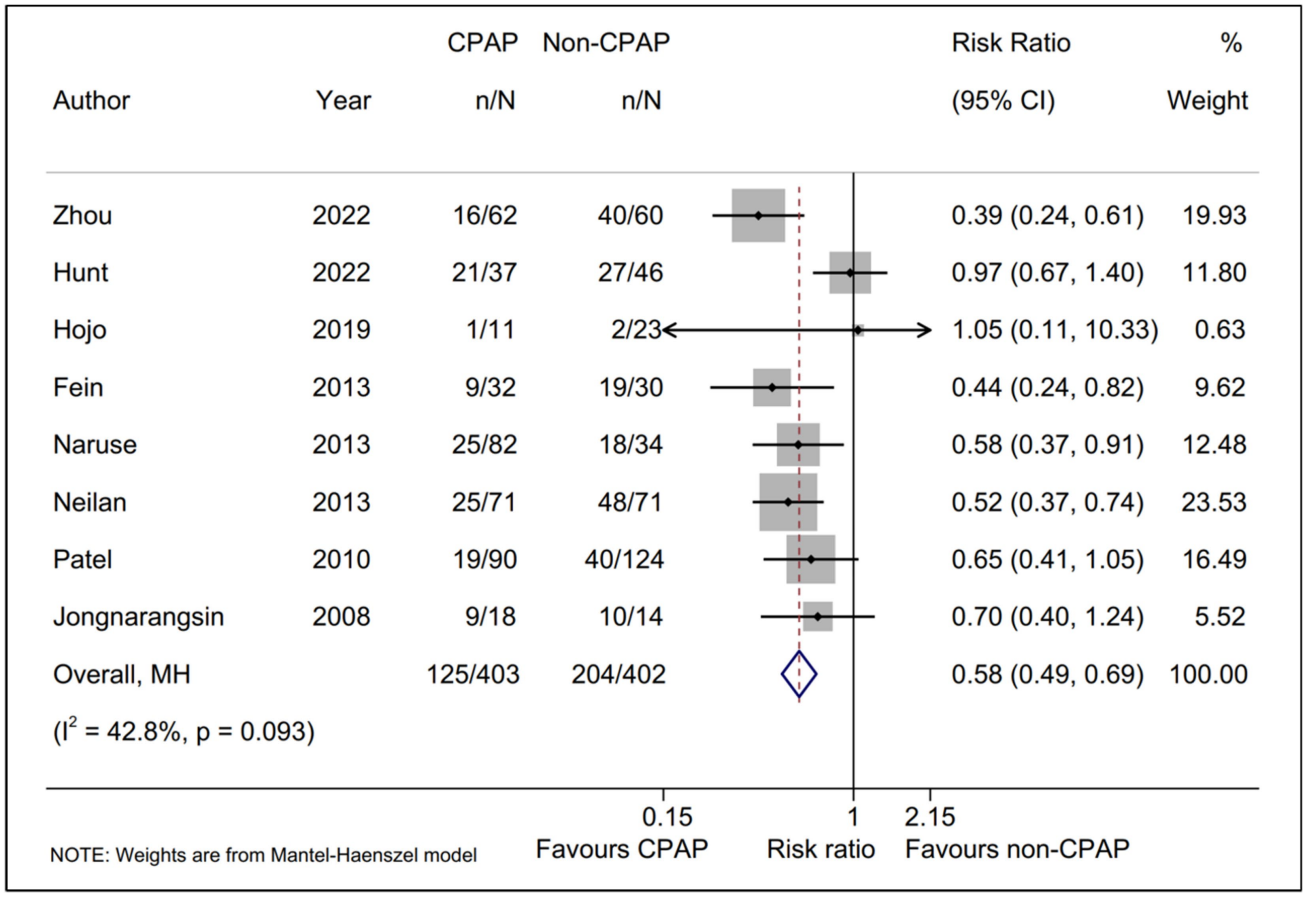
Forest plot of the AF recurrence between CPAP group and non-CPAP group. Comparison of the rate of AF recurrence between CPAP group and non-CPAP group. CPAP, continuous positive airway pressure; AF, atrial fibrillation.

A total of eight studies (15, 16, 18–23), including 805 AF patients with OSA underwent ablation in our meta-analysis, reported the AF recurrence between CPAP group and non-CPAP group. Compared with non-CPAP treatment group, CPAP treatment group was statistically associated with a lower AF recurrence rate (RR, 0.58; 95% CI, 0.49–0.69; *p* = 0.000; I^2^ = 42.80%; Figure 2) using a fixed-effect model. Meanwhile, we conducted sensitivity analysis and found that no significant change, ranging from 0.53 (95% CI, 0.44–0.64) to 0.63 (95% CI, 0.52–0.76), in the overall combined proportion, indicated that the combined proportion and heterogeneity could not be dominated via a single study (Supplementary Figure S1). Additionally, publication bias was not displayed with Egger’s test (*p* = 0.939).

Moreover, TSA was performed to evaluate whether there was a relatively adequate power for comparing the AF recurrence between CPAP group and non-CPAP group. The result suggested that the actual sample size (805) was more than the RIS (relative risk reduction = 30%; RIS = 629), and the cumulative Z curve crossed the conventional boundary, as well as the trial sequential alpha spending monitoring boundary. All TSA results revealed that there was the firm evidence favoring CPAP treatment group for the AF recurrence rate (Supplementary Figure S2).

### 3.3. The subgroup analysis of AF recurrence between CPAP group and non-CPAP group

**Figure 3:**
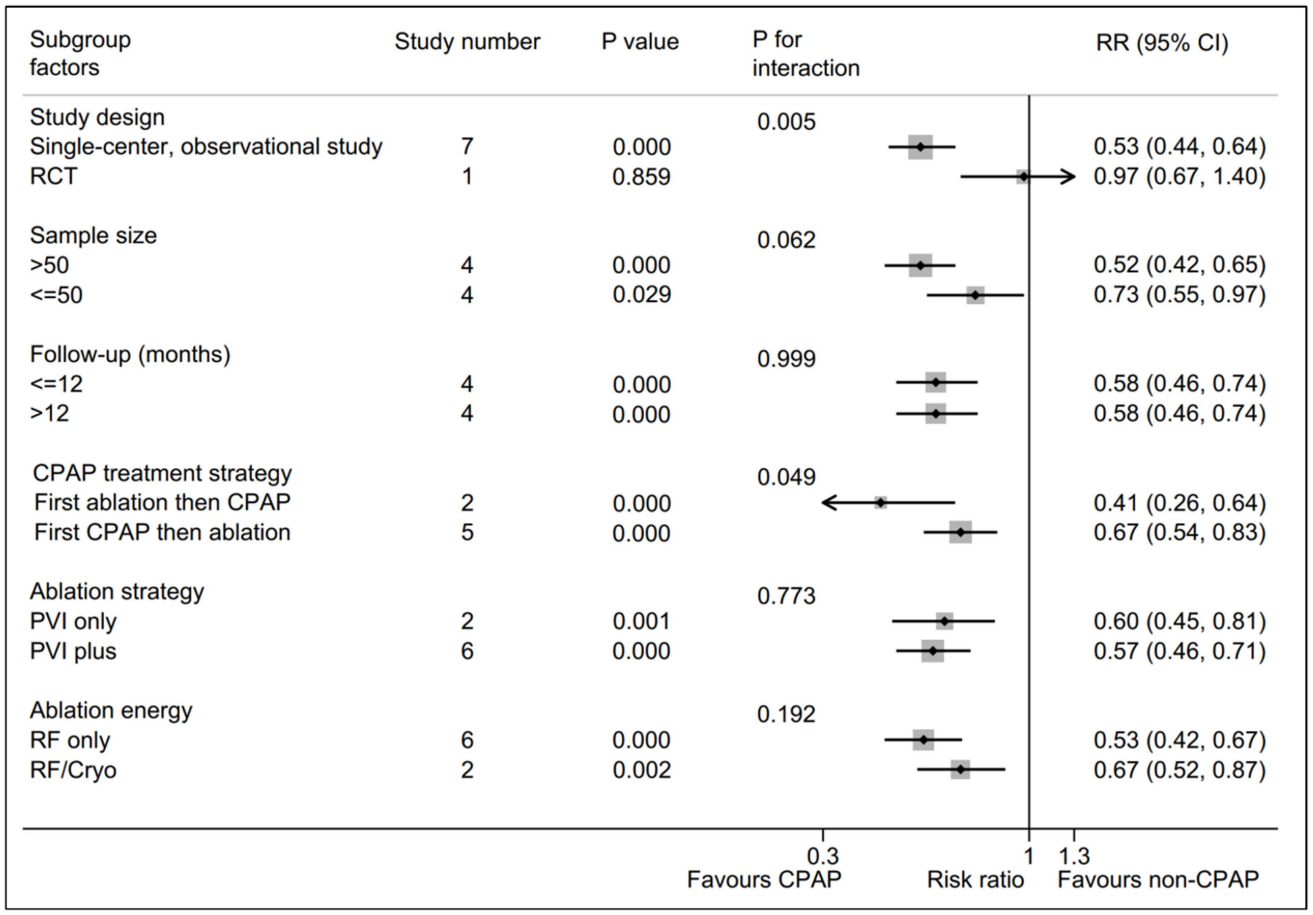
Forest plot of subgroup analysis for the AF recurrence between CPAP group and non-CPAP group. Subgroup analysis of the rate of AF recurrence between CPAP group and non-CPAP group. CPAP, continuous positive airway pressure; AF, atrial fibrillation.

A total of six subgroup factors for the AF recurrence was identified for the subgroup analysis, and the results were shown in Figure 3 and Supplementary Table S3. A statistically significant intervention-covariate interaction was identified in study design subgroup, including single-center, observational study (RR = 0.53; 95% CI, 0.44–0.64; *p* = 0.000) and RCT (RR = 0.97; 95% CI, 0.67–1.40; *p* = 0.859) with *p* = 0.005 for interaction. Meanwhile, a borderline statistically significant intervention-covariate interaction was screened in CPAP treatment strategy subgroup, including first ablation then CPAP (RR = 0.41; 95% CI, 0.26–0.64; *p* = 0.000) and first CPAP then ablation (RR = 0.67; 95% CI, 0.54–0.83; *p* = 0.000) with *p* = 0.049 for interaction. Additionally, the consistent trend with the pooled results was shown in the remaining four subgroup analysis, respectively. Interestingly, the potentially significant trend for intervention-covariate interaction were identified in sample size subgroup with *p* = 0.062.

**Figure 4:**
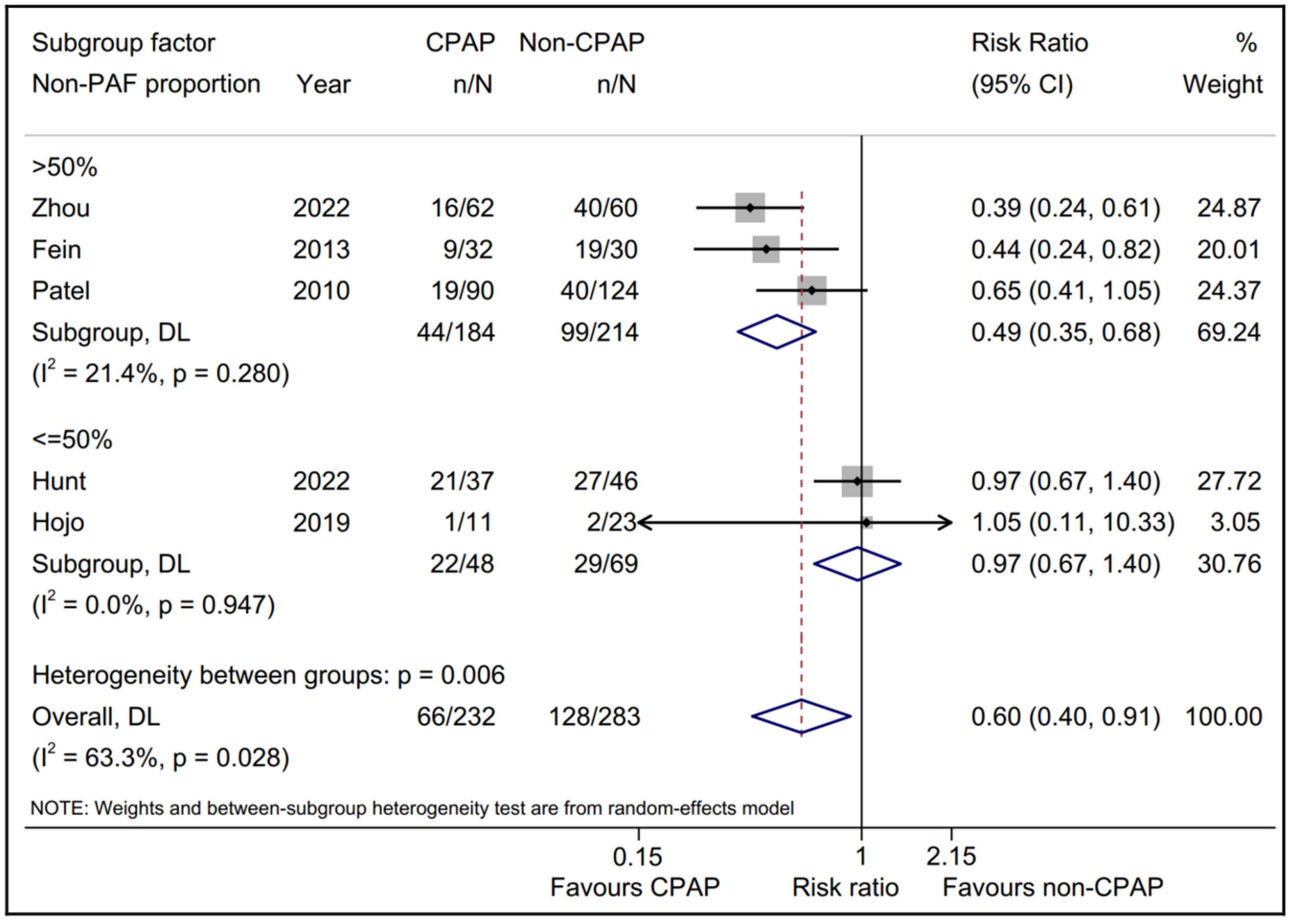
Forest plot of the non-PAF proportion subgroup analysis for the AF recurrence between CPAP group and non-CPAP group. CPAP, continuous positive airway pressure; AF, atrial fibrillation; PAF, paroxysmal atrial fibrillation.

**Figure 5:**
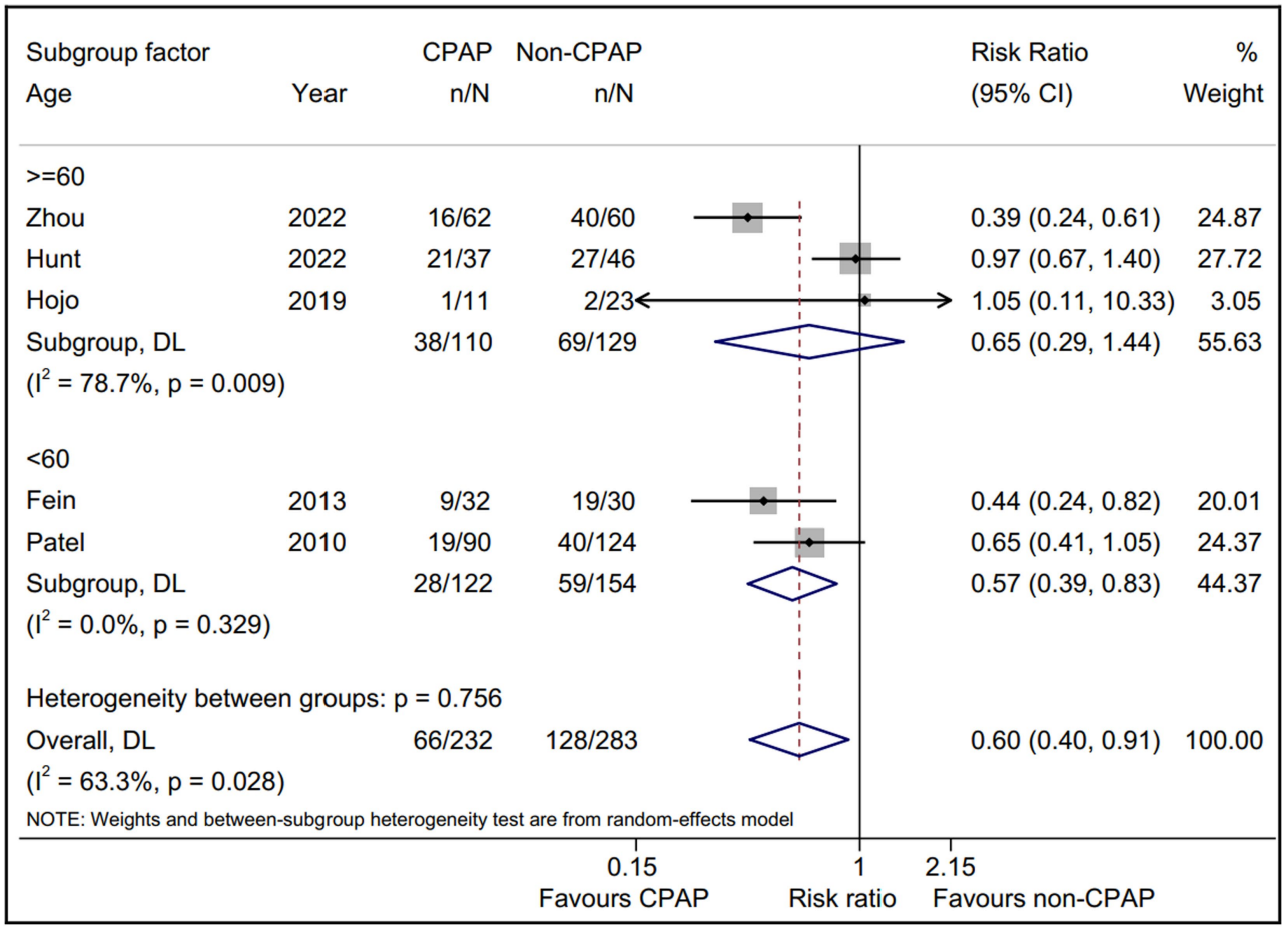
Forest plot of the age subgroup analysis for the AF recurrence between CPAP group and non-CPAP group. CPAP, continuous positive airway pressure; AF, atrial fibrillation.

Considering the limited data, we separately evaluated the non-PAF proportion subgroup (Figure 4). The pooled AF recurrence with the five eligible studies (15, 16, 18, 19, 22) using a random-effect model was consistent with the pooled result from all eligible studies (15, 16, 18–23). Interestingly, a statistically significant intervention-covariate interaction for AF recurrence was identified in non-PAF proportion subgroup, including >50% subgroup (RR = 0.49; 95% CI, 0.35–0.68; *p* = 0.000) and ≤ 50% subgroup (RR = 0.97; 95% CI, 0.67–1.40; *p* = 0.866) with *p* = 0.006 for interaction. Meanwhile, the five eligible studies (15, 16, 18, 19, 22) reported the age between CPAP and non-CPAP group, and we found that compared with non-CPAP group, the AF recurrence of CPAP group was significantly decreased in <60 years subgroup (RR = 0.57; 95% CI, 0.39–0.83; *p* = 0.003) and it was not in ≥60 years subgroup (Figure 5).

## 4. Discussion

We comprehensively enrolled a total of 1,231 AF patients with OSA (628 in the CPAP therapy group and 603 in non-CPAP therapy group) from eight clinical studies. To the best of our knowledge, this study might be the first registered meta-analysis to compare the AF recurrence for AF patients with OSA underwent ablation between CPAP therapy group and non-CPAP therapy group. The main findings are as follows: (1) CPAP therapy may be an effective strategy on reduction of AF recurrence after ablation for AF patients with OSA; (2) The CPAP treatment strategy and the non-PAF proportion might be the potential determinants of outcome impact on AF recurrence for AF patients with OSA after ablation.

A significantly epidemiological association was presented between OSA and AF. The scientific statement from American Heart Association on the OSA and cardiovascular disease have highlighted that OSA is an predominantly independent and modifiable risk factor in the initiation and progression of AF (24). West et al. (25) even reported that the AF risk was expected to increase up to 3.31 among the individuals with severe OSA. At present, the latest ESC guideline for AF management suggested that radiofrequency ablation therapy has been considered as an effective strategy for rhythm control in symptomatic and drug-refractory AF patients (2). Whereas, challenges remain on the AF patients with multiple comorbidities, especially with the underrecognized and undertreated OSA. A meta-analysis with a total of 4,572 AF patients underwent catheter ablation conducted by Congrete et al. (14) reported that the AF recurrence rate in patients with OSA was as high as 1.70 in comparison with the patients without OSA, sugggesting that the OSA could significantly impair the effective of AF ablation.

Multiple abnormal pathophysiologies underlying the OSA were directly or indirectly responsible for the development and progression of AF, including inflammation, oxidative stress, vascular endothelia dysfunction, and sympathetic overactivity. OSA was characterized with nocturnal hypoxemia and hypercapnia due to the repeated upper airway collapse during sleep, which could markly promote the activation of infalmmation, oxidative stress, and vascular endothelia dysfunction, subsequently causing to the atrial electrical and structural remodeling, ultimately leading to the AF episode (26, 27). Moreover, acute apneic episodes in OSA could result in sympathetic overactivity, shortening atrial refractoriness, increasing triggered activity and promoting the AF initiation (28, 29).

CPAP has been reported to be an effective alternation to markly improve and correct the abnormal pathophysiologies underlying the OSA. Accumulated evidence indicated that the CPAP therapy could significantly reduce the AF episodes in OSA patients. A prospective cohort study conducted by Varga et al. (12) including ninety-three patients with OSA suggested that the prevalence of cardiac arrhythmias (such as AF) was significantly declined with three-month CPAP therapy (*p* = 0.03). A randomized controlled trial (NCT01165710) including 10,132 AF patients with two-year follow-up also indicated that the OSA patients underwent CPAP treatment were less likely to the progression to permanent AF compared with OSA patients without CPAP therapy (hazard ratio = 0.66; 95% CI, 0.46–0.94; *p* = 0.021) (13). However, the effective of the CPAP on AF patients with OSA after ablation is still controversial. An previous meta-analysis on the efficacy of AF patients with OSA underwent CPAP therapy after ablation highlighted that the patients with OSA not undergoing CPAP therapy had a 57% greater risk of AF recurrence compared with that undergoing CPAP therapy (30). Whereas, a recent randomized controlled trial showed the different outcomes. In paroxysmal AF patients with OSA, CPAP treatment failed to further reduce the risk of AF recurrence after ablation, and PVI could significantly reduced the AF burden in OSA patients without any statistical difference between CPAP therapy and non-CPAP therapy group (16). Therefore, we perfomed this meta-analysis to clarify the role of CPAP on the AF patients with OSA after ablation.

Our results suggested that compared with non-CPAP treatment group, CPAP treatment group was significantly associated with a lower AF recurrence rate, indicating that the CPAP could reduce the AF recurrence after ablation in AF patients with OSA. Sensitivity analysis and Egger’s test revealed that our results were relatively robust. Meanwhile, TSA result also indicated the firm evidence favoring CPAP group for the AF recurrence risk. This result further supported the pooled results.

Subgroup analysis was conducted to identify the potential determinants for the AF recurrence rate between the CPAP therapy group and non-CPAP therapy group for the AF patients with OSA. A statistically significant intervention-covariate interaction was identified in study design subgroup, suggesting that the previous observational studies showed a benefit outcome in CPAP group and RCT did not. Whereas, only one RCT was eligible in our meta-analysis, which could lead to potential bias due to limitated sample size (16). Noteworthily, another statistically significant interaction was identified in non-PAF proportion subgroup, revealing that the benefit for CPAP could be achieved in >50% subgroup (RR = 0.49; *p* = 0.000) while it could not in ≤50% subgroup (RR = 0.97; *p* = 0.866). The results suggested that the higher proportion of non-PAF was associated with the more benefits for CPAP on the AF patients with OSA after ablation, which could partly account for the negative result of the only RCT without non-PAF patients (16). Importantly, this subgroup result provided a basis for a RCT to further evaluate the role of AF type on the effect of CPAP in AF patients with OSA after ablation.

In addition, a borderline statistically significance (*p* = 0.049 for interaction) was presented between first ablation then CPAP subgroup (RR = 0.41) and first CPAP then ablation subgroup (RR = 0.67), which indicated that first ablation then CPAP strategy might be superior to first CPAP then ablation strategy for the AF patients with OSA after ablation. The potential explanations were as follows. CPAP was reported to markedly improve oxidative stress, inflammation and sympathetic overactivity in OSA, which means that it could suppress the trigger activities from atrium to a certain extent (26). Therefore, we made a reasonable speculation that the potential focal activities might be masked with CPAP treatment prior to ablation, leading to a incomplete elimiatation of the ectopic focus within the atrium during ablation, ultimately causing that first CPAP then ablation strategy showed a inferior results in comparison with first ablation then CPAP strategy. Similarly, this subgroup result might be expected to be a preliminary basis to clarify the role of CPAP treatment strategy via a prospective RCT with large cohorts.

Increasing studies have demonstrated that a higher volume for intervention group was significantly associated with a better outcome, which indicated the “practice makes perfect” principle (31, 32). Similarly, a potentially significant trend (*p* = 0.062) was displayed in sample size subgroup with, suggesting that the larger sample size in CPAP treatment group tended to be associated with the lower AF recurrence. Moreover, the remaining three subgroups, including follow-up time, ablation strategy, and ablation energy, showed the consistent trend to the pooled results without significantly statistical interaction, respectively. These subgroup results suggested that the benefits of CPAP on AF patients with OSA after ablation were not affected by the three subgroup items.

Age is an independent risk factor for the AF progress and prognosis, and aging is reported to be associated with a high AF recurrence after ablation therapy (33, 34). Whereas, no data to date revealed the effect of age on the AF recurrence with/without CPAP therapy for OSA patients after ablation treatment. In this study, we found that the young AF patients with OSA (<60 years) could acquire benefits from CPAP therapy, and the elderly ones (≥60 years) did not, further suggesting that aging might play a detrimental role on the CPAP therapy for AF patients with OSA after ablation. This subgroup results also indicated that the young AF patients with OSA underwent ablation might be a more suitable subjects with CPAP therapy, which was expected to provide a theoretical basis for personalized treatment AF combined OSA.

## 5. Limitations

There are several limitations in our study. First, in the most of our eligible studies, the diagnosis of AF recurrence was based on the periodic and occasional ECG or 24 h Holter recording, as well as the AF symptoms, whereas only one study used the continuous monitoring of AF burden with a loop recorder for the diagnosis of AF recurrence. Therefore, the asymptomatic AF recurrence might been easily ignored, leading to potentially underestimate the AF recurrence and fail to accurately evaluate the effective of CPAP on the AF patients with OSA after ablation. More studies with a loop recorder should be performed to further assess the role of CPAP. Second, the AF type was also an key factor determined the ablation prognosis. Whereas, in this study, we found that the higher proportion of non-PAF and the more benefits with CPAP on the AF patients with OSA after ablation. Due to limitation of eligible studies and available data, the result should be interpreted with caution until confirmed in larger studies. Third, our study belongs to the meta-analysis, and some potential biases might affect our results. Whereas, sensitivity analysis and publication bias test (such as Egger’s test) both suggested our robust results. Meanwhile, TSA result also provide the firm evidence to support the pooled results. Finally, only one RCT with limitated sample size was eligible in this study, which may lead to the possible bias and restrict us to draw a substantial conclusion. Therefore, more RCTs with large sample size are needed for further demonstrating these results.

## 6. Conclusion

Our study suggests that CPAP therapy might be an effective strategy on reduction of AF recurrence after ablation for AF patients with OSA. The CPAP treatment strategy and the non-PAF proportion might be the possible determinants on AF recurrence for AF patients with OSA after ablation. More RCTs with large sample size should be designed and performed to further demonstrate our results.

## Data availability statement

The original contributions presented in the study are included in the article/Supplementary material, further inquiries can be directed to the corresponding authors.

## Author contributions

FL: Data curation, Methodology, Writing – original draft. C-JH: Data curation, Methodology, Writing – review & editing. C-HD: Writing – review & editing. R-XW: Validation, Writing – review & editing. HL: Conceptualization, Validation, Writing – review & editing.
