# Supplementary material for: Continuous positive airway pressure therapy might be an effective strategy on reduction of atrial fibrillation recurrence after ablation in patients with obstructive sleep apnea: insights from the pooled studies
Source: Front Neurol. 2023 Nov 9;14:1269945. doi: 10.3389/fneur.2023.1269945 (PMC10665895; doi:10.3389/fneur.2023.1269945)
Supplement: Supplementary file 1 [file Data_Sheet_1.docx]

**Supplementary Table 1.** Quality assessment of eligible studies according to the Newcastle-Ottawa Quality Assessment Scale (NOS)

| First author | Year | Selection |  |  |  | Comparability | Outcome |  |  | Total stars |
| --- | --- | --- | --- | --- | --- | --- | --- | --- | --- | --- |
|  |  | Representativeness of the exposed cohort | Selection of the nonexposed cohort | Ascertainment of exposure | Demonstration that outcome of interest was not present at start of study | Comparability of cohorts on the basis  of the design or analysis | Assessment of outcome | Was follow-up long enough for outcomes to occur | Adequacy of follow-up of cohorts |  |
| Zhou^[15]^ | 2022 | **☆** | **☆** | **☆** | **☆** | **☆☆** | **☆** | **☆** | **☆** | 9 |
| Hojo^[18]^ | 2019 | **☆** | **☆** | **☆** | **☆** | **☆☆** | **☆** | **☆** | **☆** | 9 |
| Fein^[19]^ | 2013 | **☆** | **☆** | **☆** | **☆** | **☆☆** | **☆** | **☆** | **☆** | 9 |
| Naruse^[20]^ | 2013 | **☆** | **☆** | **☆** | **☆** | **☆☆** | **☆** | **☆** | **☆** | 9 |
| Neilan^[21]^ | 2013 | **☆** | **☆** | **☆** | **☆** | **☆☆** | **☆** | **☆** | **☆** | 9 |
| Patel^[22]^ | 2010 | **☆** | **☆** | **☆** | **☆** | **☆☆** | **☆** | **☆** | **☆** | 9 |
| Jongnarangsin^[23]^ | 2008 | **☆** | **☆** | **☆** | **☆** | **☆☆** | **☆** | **☆** |  | 8 |

**Supplementary Table 2.** Quality assessment for randomized clinical trials according to the Cochrane risk of bias assessment tool

| First author | Year | Random sequence generation (selection bias) | Allocation concealment (selection bias) | Blinding of participants and personnel (performance bias) | Blinding of outcome assessment (detection bias) | Incomplete outcome data (attrition bias) | Selective reporting (reporting bias) | Other bias |
| --- | --- | --- | --- | --- | --- | --- | --- | --- |
| Hunt^[16]^ | 2022 | **U** | **U** | **H** | **U** | **L** | **L** | **L** |

L: Low risk of bias; H: High risk of bias; U: Uncertain.

**Supplementary Table 3.** Subgroup analysis of AF recurrence between CPAP group and non-CPAP group

| **Subgroup factors** | **Numbers of Study** | **RR (95%CI)** | **I^2^ (%)** | ***P* value** | ***P* for interaction** |
| --- | --- | --- | --- | --- | --- |
| Study design |  |  |  |  | 0.005 |
| Single-center, observational study | 7 | 0.53 (0.44, 0.64) | 0.00 | 0.000 |  |
| RCT | 1 | 0.97 (0.67, 1.40) | - | 0.859 |  |
| Sample size |  |  |  |  | 0.062 |
| >50 | 4 | 0.52 (0.42, 0.65) | 0.00 | 0.000 |  |
| ≤50 | 4 | 0.73 (0.55, 0.97) | 37.40 | 0.029 |  |
| Follow-up (months) |  |  |  |  | 0.999 |
| ≤12 | 4 | 0.58 (0.46, 0.74) | 73.60 | 0.000 |  |
| >12 | 4 | 0.58 (0.46, 0.74) | 0.00 | 0.000 |  |
| CPAP treatment strategy |  |  |  |  | 0.049 |
| First ablation then CPAP | 2 | 0.41 (0.26, 0.64) | 0.00 | 0.000 |  |
| First CPAP then ablation | 5 | 0.67 (0.54, 0.83) | 32.20 | 0.000 |  |
| Ablation strategy |  |  |  |  | 0.773 |
| PVI only | 2 | 0.60 (0.45, 0.81) | 89.80 | 0.001 |  |
| PVI plus | 6 | 0.57 (0.46, 0.71) | 0.00 | 0.000 |  |
| Ablation energy |  |  |  |  | 0.192 |
| RF only | 6 | 0.54 (0.42, 0.67) | 0.00 | 0.000 |  |
| RF/Cryo | 2 | 0.67 (0.52, 0.87) | 82.40 | 0.002 |  |

Abbreviation: AF: atrial fibrillation; CPAP: continuous positive airway pressure; RCT: randomized controlled trial; PVI: pulmonary vein isolation; RF: radiofrequency; Cryo: cryoballoon; RR: risk ratio.

**Supplementary Text 1**

## 1. Four online databases, including PubMed, Embase, Cochrane Library, and Web of Science, were searched from the establishment of the online databases up to 1 February 2023.

## 2. MeSH Terms

|  | PubMed-MeSH |
| --- | --- |
| OSA | (Sleep Apnea, Obstructive) OR (Apneas, Obstructive Sleep) OR (Obstructive Sleep Apneas) OR (Sleep Apneas, Obstructive) OR (Obstructive Sleep Apnea Syndrome) OR (Obstructive Sleep Apnea) OR (OSAHS) OR (Syndrome, Sleep Apnea, Obstructive) OR (Sleep Apnea Syndrome, Obstructive) OR (Apnea, Obstructive Sleep) OR (Sleep Apnea Hypopnea Syndrome) OR (Syndrome, Obstructive Sleep Apnea) OR (Upper Airway Resistance Sleep Apnea Syndrome) OR (Syndrome, Upper Airway Resistance, Sleep Apnea) |
| AF | (Atrial Fibrillation) OR (Atrial Fibrillations) OR (Fibrillation, Atrial) OR (Fibrillations, Atrial) OR (Auricular Fibrillation) OR (Auricular Fibrillations) OR (Fibrillation, Auricular) OR (Fibrillations, Auricular) OR (Persistent Atrial Fibrillation) OR (Atrial Fibrillation, Persistent) OR (Atrial Fibrillations, Persistent) OR (Fibrillation, Persistent Atrial) OR (Fibrillations, Persistent Atrial) OR (Persistent Atrial Fibrillations) OR (Familial Atrial Fibrillation) OR (Atrial Fibrillation, Familial) OR (Atrial Fibrillations, Familial) OR (Familial Atrial Fibrillations) OR (Fibrillation, Familial Atrial) OR (Fibrillations, Familial Atrial) OR (Paroxysmal Atrial Fibrillation) OR (Atrial Fibrillation, Paroxysmal) OR (Atrial Fibrillations, Paroxysmal) OR (Fibrillation, Paroxysmal Atrial) OR (Fibrillations, Paroxysmal Atrial) OR (Paroxysmal Atrial Fibrillations) |
| CPAP | (Continuous Positive Airway Pressure) OR (CPAP Ventilation) OR (Ventilation, CPAP) OR (Nasal Continuous Positive Airway Pressure) OR (nCPAP Ventilation) OR (Ventilation, nCPAP) OR (Airway Pressure Release Ventilation) OR (APRV Ventilation Mode) OR (APRV Ventilation Modes) OR (Ventilation Mode, APRV) OR (Ventilation Modes, APRV) OR (Biphasic Continuous Positive Airway Pressure) OR (BiPAP Biphasic Positive Airway Pressure) OR (BiPAP Bilevel Positive Airway Pressure) OR (Biphasic Positive Airway Pressure) OR (Bilevel Continuous Positive Airway Pressure) OR (Bilevel Positive Airway Pressure) |
| Recur | (Recurrence) OR (Recurrences) OR (Recrudescence) OR (Recrudescence) OR (Relapse) OR (Relapses) |
| Ablation | (Catheter Ablation) OR (Ablation, Catheter) OR (Catheter Ablation, Transvenous) OR (Transvenous Catheter Ablation) OR (Ablation, Transvenous Catheter) OR (Catheter Ablation, Electric) OR (Electrical Catheter Ablation) OR (Catheter Ablation, Electrical) OR (Ablation, Electrical Catheter) OR (Electric Catheter Ablation) OR (Ablation, Electric Catheter) OR (Ablation, Transvenous Electric) OR (Electric Ablation, Transvenous) OR (Transvenous Electric Ablation) OR (Ablation, Transvenous Electrical) OR (Electrical Ablation, Transvenous) OR (Transvenous Electrical Ablation) OR (Catheter Ablation, Radiofrequency) OR (Radiofrequency Catheter Ablation) OR (Ablation, Radiofrequency Catheter) OR (Catheter Ablation, Percutaneous) OR (Percutaneous Catheter Ablation) OR (Ablation, Percutaneous Catheter) OR (Radiofrequency Ablation) OR (Ablation, Radiofrequency) OR (Radio Frequency Ablation) OR (Ablation, Radio Frequency) OR (Radio-Frequency Ablation) OR (Ablation, Radio-Frequency) |

3. Search strategy：OSA AND AF AND CPAP AND Recur AND Ablation

|  | MeSH terms | PubMed | Embase | The Cochrane Library | Web of Science Core Collection |
| --- | --- | --- | --- | --- | --- |
| #1 | OSA | 41763 | 49902 | 6828 | 49377 |
| #2 | AF | 103982 | 160094 | 15028 | 121841 |
| #3 | CPAP | 16915 | 18290 | 5962 | 14238 |
| #4 | Recur | 945530 | 827306 | 112889 | 601076 |
| #5 | Ablation | 54357 | 58244 | 5814 | 59563 |
| #6 | #1 AND #2 AND #3 AND #4 AND #5 | 29 | 25 | 7 | 28 |

4. Several notes

(i) Note: A total of 89 articles were searched from the four online databases. Sixty articles were remained after duplicates removed, in which one is an Ongoing RCT (ID: ACTRN12616000088448).

(ii) Note: We provided the Web address of the Ongoing RCT (ID: ACTRN12616000088448)

<https://www.cochranelibrary.com/central/doi/10.1002/central/CN-02439973/full> OR https://trialsearch.who.int/Trial2.aspx?TrialID=ACTRN12616000088448

**Supporting materials**

**Full-text articles excluded (n = 9)**

(1) Cardioversion or other intervention 5 articles

1. 2019-The Effect of Positive Airway Pressure Treatment of Obstructive and Central Sleep Apnea on the Recurrence of Atrial Fibrillation/Flutter Postintervention.

2. 2019-3.22-The impact of continuous positive airway pressure treatment on the recurrence of atrial fibrillation post cardioversion A randomized controlled trial.

3. 2003-23.6-Obstructive sleep apnea and the recurrence of atrial fibrillation.

4.2013-8.3-Obstructive sleep apnea in patients with typical atrial flutter prevalence and impact on arrhythmia control outcome.

5. 2019-3.22-The impact of continuous positive airway pressure treatment on the recurrence of atrial fibrillation post cardioversion A randomized controlled trial.

(2) Meta-analysis 3 article

1. 2018-2.1-Effect of obstructive sleep apnea and its treatment of atrial fibrillation recurrence after radiofrequency catheter ablation A meta-analysis.

2.2014-4.04-Efficacy of catheter ablation of atrial fibrillation in patients with OSA with and without CPAP treatment a meta-analysis.

3. 2018-4.8-Treating obstructive sleep apnea with continuous positive airway pressure reduces risk of recurrent atrial fibrillation after catheter ablation: a meta-analysis. Sleep Med. 2018;46:5-11.

(3) Ongoing RCT 1 article

1. An ongoing RCT (ID: ACTRN12616000088448) <https://www.cochranelibrary.com/central/doi/10.1002/central/CN-02439973/full> OR https://trialsearch.who.int/Trial2.aspx?TrialID=ACTRN12616000088448
