# Supplementary material for: Continuous positive airway pressure therapy might be an effective strategy on reduction of atrial fibrillation recurrence after ablation in patients with obstructive sleep apnea: insights from the pooled studies
Source: Front Neurol. 2023 Nov 9;14:1269945. doi: 10.3389/fneur.2023.1269945 (PMC10665895; doi:10.3389/fneur.2023.1269945)
Supplement: Supplementary file 2 [file Data_Sheet_2.doc]

**Supplementary Figure S1**


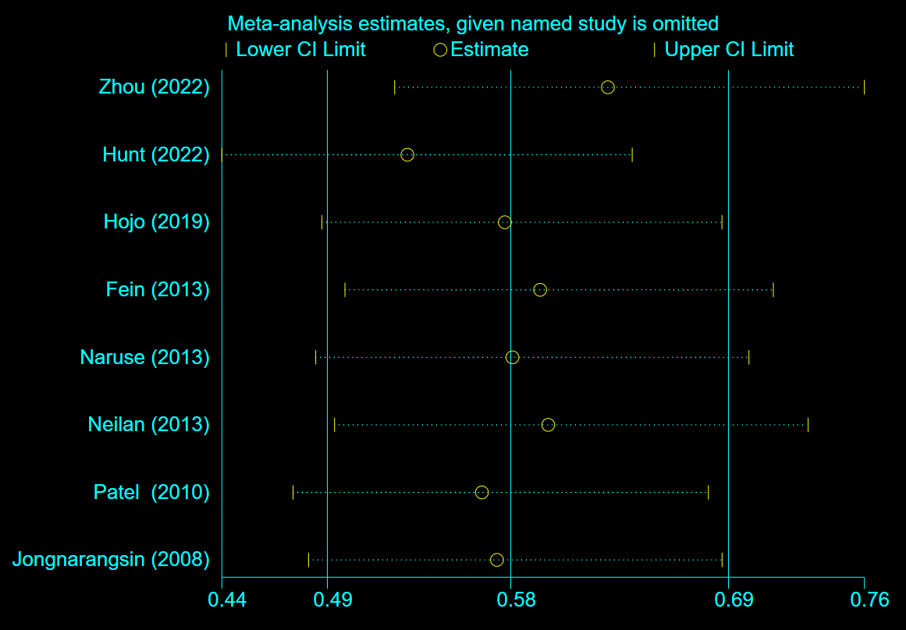


**Supplementary Figure S1.** Sensitivity analysis with sequentially omitting one study at a time for the AF recurrence between CPAP group and non-CPAP group. Sensitivity analysis showed no significant change in the overall combined proportion. AF: atrial fibrillation; CPAP: continuous positive airway pressure.

**Supplementary Figure S2**

**
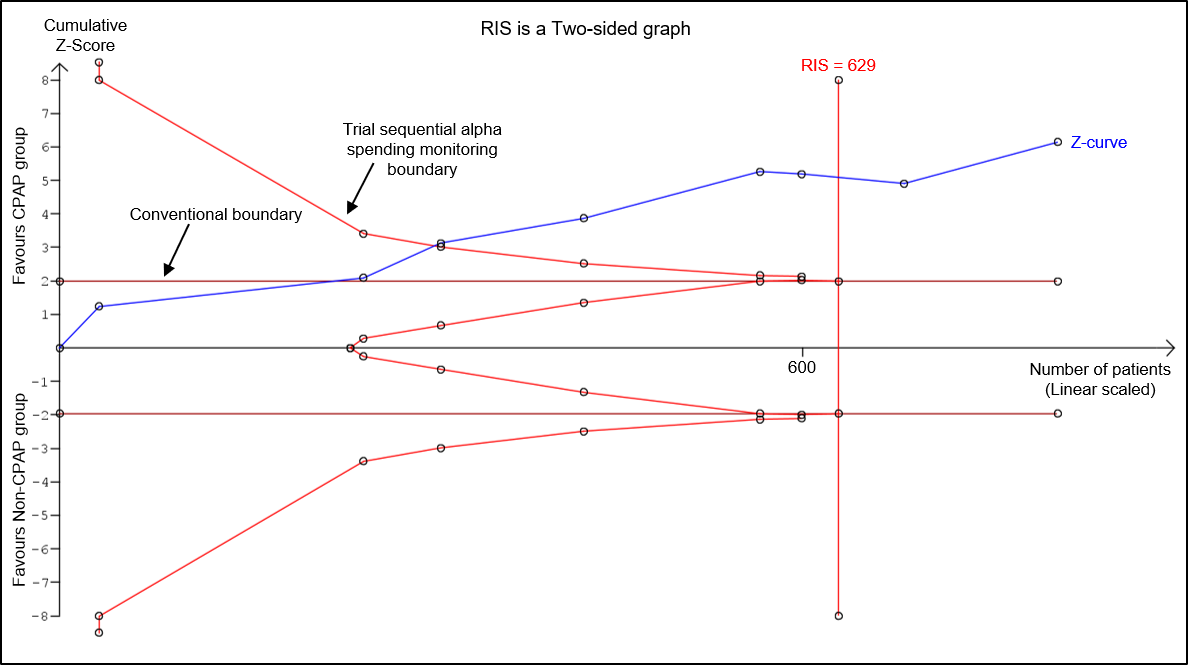
**

**Supplementary Figure S2.** TSA for the AF recurrence between CPAP group and non-CPAP group. The result suggested that the actual sample size (805) was more than the RIS (relative risk reduction, RRR=30%; RIS=629), and the cumulative Z curve had crossed both the conventional boundary and the trial sequential alpha spending monitoring boundary. AF: atrial fibrillation; CPAP: continuous positive airway pressure; TSA: trial sequential analysis; RRR: relative risk reduction; RIS: required information size.
